# Workplace violence towards nurses in Hong Kong: prevalence and correlates

**DOI:** 10.1186/s12889-017-4112-3

**Published:** 2017-02-14

**Authors:** Teris Cheung, Paul S. F. Yip

**Affiliations:** 1 School of Nursing, Hong Kong Polytechnic University, Hung Hom, Hong Kong; 2 Centre for Suicide Research and Prevention, University of Hong Kong, Pok Fu Lam, Hong Kong

**Keywords:** Cross-sectional, DASS_21_, Nurses, Occupational hazards, Workplace violence, Public health, Hong Kong

## Abstract

**Background:**

Nurses are especially vulnerable to violent and other forms of aggression in the workplace. Nonetheless, few population-based studies of workplace violence have been undertaken among working-age nurse professionals in Hong Kong in the last decade.

**Methods:**

The study estimates the prevalence and examines the socio-economic and psychological correlates of workplace violence (WPV) among professional nurses in Hong Kong. The study uses a cross-sectional survey design. Multivariate logistic regression examines the weighted prevalence rates of WPV and its associated factors for a population of nurses.

**Results:**

A total of 850 nurses participated in the study. 44.6% had experienced WPV in the preceding year. Male nurses reported more WPV than their female counterparts. The most common forms of WPV were verbal abuse/bullying (39.2%), then physical assault (22.7%) and sexual harassment (1.1%). The most common perpetrators of WPV were patients (36.6%) and their relatives (17.5%), followed by colleagues (7.7%) and supervisors (6.3%). Clinical position, shift work, job satisfaction, recent disturbances with colleagues, deliberate self-harm (DSH) and symptoms of anxiety were significantly correlated with WPV for nurses.

**Conclusions:**

WPV remains a significant concern for healthcare worldwide. Hong Kong’s local health authority should put in place a raft of zero-tolerance measures to prevent WPV in healthcare settings.

## Background

The occupational health and safety hazard of workplace violence (WPV) [1] has been the subject of extensive research at international level [2–5]. The WHO define WPV as “incidents where staff are abused, threatened, or assaulted in circumstances related to their work, including commuting to and from work, involving an explicit or implicit challenge to their safety, well-being or health” [6]. International studies further identify the medical profession, particularly nursing, as one of the occupations at elevated risk of WPV [7, 8]. WPV may be broken down into physical, psychological or sexual violence (or harassment) [9, 10]. Physical violence is defined as an intentional behaviour aiming to harm another person physically [11]. Psychological violence aims at psychological damage to the victim and is often accompanied by other types of violence [12, 13]. Sexual violence (or harassment) takes verbal and physical forms, and can be construed as unwanted, unreciprocated or unwelcome behaviour of a sexual nature tending to humiliate, threaten or embarrass [9, 11].

International studies find the prevalence of WPV against nurses in hospital settings varies from 10% to 50%, in one case going as high as 87% [14–16]. The studies were conducted in Western countries. There are few studies examining the prevalence of WPV and its correlates in Asia [17]. Asian studies are also limited by small sample size or low response rates [17]. Some research limits consideration of WPV to the past 12 months, which may subject results to recall bias. This could lead to underestimates of the scale of WPV.

A decade ago, Kwok et al. [18] examined the prevalence of WPV on 420 nurses in a university teaching hospital in Hong Kong. Results showed that 76% (*n* = 320) nurses experienced verbal abuse (73%), bullying (45%), physical abuse (18%), and sexual harassment (12%). A vast majority of those (84%) experiencing WPV confide in friends, family members or colleagues. Others (42%) ignored the incident. Older research also finds that verbal abuse is apparently the most common form of WPV in healthcare settings in Hong Kong; the key perpetrators are mostly patients and their relatives [19]. Colleagues and supervisors have also been identified as committing WPV [11].

Recent epidemiological data (*n* = 588) suggest that 71.9% of nurses in China reported non-physical and 7.8% physical violence in the year before the study [20]. The violent parties were patients (93.5%) and patients’ relatives (82%). Inexperienced nurses were more likely to report physical/non-physical violence. Graduate-level nurses again reported WPV proportionately more than non-graduates. Nurses on rotating shift duty were 3.67 times (95% CI 1.28–10.55) more likely to experience physical violence than on fixed day duty. Higher anxiety levels were significantly associated with violence. An Australian study (*n* = 273) specifically found that nurses working morning shifts were more likely to experience bullying than other shift workers. Younger nurses with less than 15 years of experience were more likely to report physical assault than those with 25 years or more years of experience [21].

A cross-sectional study (*n* = 521) in Taiwan reported similar findings. Specifically, nurses being under 30 or working nights increased the odds of verbal abuse, while bullying also correlated with anxiety. An association held between physical violence and reporters holding a bachelor’s degree [11]. The prevalence of verbal abuse and physical violence came in at 51.4% and 19.6%, respectively.

The under-reporting of incidents of WPV is not uncommon in the healthcare profession [22]. Nurses may stay silent because they fear retaliation; because they have not sustained injuries; because reporting procedures are complex, or because they feel unsupported by management [19]. Many purportedly consider WPV an occupational hazard [23]. Emergency departments, psychiatric units and intensive care units emerge as the most common sites of WPV [24–26].

Although most WPV is classified as non-physical psychological violence, some victims report post-traumatic stress symptoms after violent episodes [11]. WPV may negatively affect nurses’ physical and psychological well-being, reducing job satisfaction, staff morale and job performance [20]. WPV may lead to absenteeism, bring bad publicity onto healthcare, and precipitate long-term negative physical or psychological effects [2]. In consequence, it can jeopardize the quality of patient care [27–29].

Despite well-known work highlighting the negative consequences of WPV on victims locally and internationally, it appears that no research on WPV towards nurses has been undertaken in Hong Kong in the healthcare setting in the last decade. This study sets out to examine the prevalence of WPV and its socio-demographic correlates among Hong Kong nurses. The investigator will also identify the most common forms of WPV in healthcare settings and their perpetrators. The nursing implications of the phenomenon of WPV will also be discussed at the end of the paper.

## Methods

### Aim

This paper forms part of a larger survey-based study of nurses’ mental health. Specifically, it sets out to examine the weighted prevalence of WPV among nurses in the context of a statement of socio-demographic characteristics of nurses working in healthcare setting in Hong Kong.

### Study design

This study used a cross-sectional survey design. It took account of existing nursing literature on mental health in drawing up a nine-section, web-based survey, administered by nurses to themselves.

### Participants

A mass invitation email was delivered to members of the Association of Hong Kong Nursing Staff (AHKNS) the biggest nursing association in Hong Kong. The AHKNS represents over 50% (*n* =22,000) of all qualified nurses registered with the Hong Kong Nursing Council (AHKNS, 2013). All such nurses, of both genders, aged between 18 and 65, currently working full-time in any healthcare setting were invited to participate in this study. Non-readers of Chinese were excluded on account of the survey being in that language. Since we used a web-based survey, we could only send our survey to those AHKNS members registered with an email account (*n* = 16,082). Data collection spanned a four-week period from October 2013 to November 2013. The AHKNS sent a follow-up message two weeks after the first mailing of the instrument to stimulate responses.

### Instrument

#### Data collection tools and measurements

All questions on WPV were derived from the ***“Workplace violence in the health sector country case studies research instruments survey questionnaires***” (English version) as set out by an ILO/ICN/WHO/PSI project. The instrument was translated into Chinese, and we invited 8 mental health experts in Hong Kong to evaluate its content validity, including the appropriateness of the translation and comprehensibility of the questions asked. Confirmation of test-retest reliability (0.85) and consistency was assessed for the survey with 20 nurses in four regional hospitals. A retest was performed two weeks later. The questions were then back-translated to English to verify the accuracy of the Chinese version.

Definitions of workplace violence incorporated the original definition framed by the World Health Organization, as shown in the box below:

**Table:** 

Verbal abuse – vulgarity, insult, sniggering Bullying – unreasonable workloads or shifts Physical abuse – physical assault, slapping, kicking, other forms of physical affront Sexual harassment – verbal remarks of a sexual nature, lewd gestures or hints, any form of sexualized physical action	

The questionnaire posed the following ten questions:

Have you encountered any workplace violence in the past 12 months? (yes/no).

What was the nature of the violence?

(1: verbal abuse/bullying; 2: physical assault; 3: sexual abuse/assault)

Who was responsible for the violence? (1: patients; 2: relatives; 3: colleagues; 4: supervisors; 5: others).

How did you respond to the violence?

(1: reported it to senior staff member; 2: told the perpetrator to stop; 3: ignored the incident; 4: switched job; 5: told friends/relatives; 6: told a colleague; 7: sought help from union; 8: sought counselling; 9: completed incident report form; 10: prosecuted; 11: made a compensation claim; 12: other).

Did you sustain any physical injury needing treatment by medical personnel? (yes/no)

Do you think the violence could have been prevented? (yes/no)

How would you rate the impact of the violence on your mental health? (1: not at all to 10: very severe).

Questions 8 to 10 asked whether participants had any history of consulting psychiatrists and elicited their psychiatric diagnoses (if any) following on from the WPV episodes.

Socio-demographic and other work-related information was obtained via a self-reported self-administrative web-based survey. Depression, anxiety and symptoms of stress were measured by Lovibond and Lovibond’s short version of the Depression Anxiety Stress Scale (DASS) [23].

#### Depression anxiety stress scale 21 (DASS-21)

The paper reports weighted prevalence for depression, anxiety and symptoms of stress, and their correlates, as measured by a short version of Depression Anxiety and Stress Scale (DASS_21_) [23]. This Depression Anxiety Stress Scale 21 (DASS_21_), has been validated as a reliable self-administered psychological instrument consisting of 21 items in three domains. Each domain comprises seven items assessing three dimensions of mental health symptoms: depression, anxiety and stress. Respondents were required to indicate the presence of these symptom(s) over the past week on a 4-point Likert scale scoring from 0–3 (0: did not apply at all over the last week, 1: applied to some degree, or some of the time; 2: applied a considerable degree, or a good part of time; 3: applied very much or most of the time). The more severe the symptoms in each dimension, the higher the subscale scores. The instrument is frequently used in clinical and non-clinical samples [30–34] and possesses well-established psychometric properties in reliably measuring depression, anxiety and stress (at a Cronbach’s alpha 0.91, 0.84 and 0.90, respectively). The scale credibly differentiates between depression, anxiety and stress [30, 35–38]. Our study used the validated Chinese translation of the DASS_21_ as participants were predominantly ethnically Chinese. Scores from each dimension were summed up and categorized as “normal”, “mild”, “moderate”, “severe” and “extremely severe”, according to the DASS manual [30].

#### Statistical analysis

Whether or not participants had encountered with Workplace Violence (WPV) was coded as a dichotomous response (yes/no). Responses relating to the perceived impact of WPV on participants’ mental health were coded into three categories (mild, moderate and severe) before being entered into binary logistic regression. The prevalence of WPV was examined and presented in terms of frequency and the proportion of those encountering it (%). Prevalence estimates (%) were presented at 95% confidence intervals (CI) calculated from the SE. Bivariate and multivariate analyses measured the strength of associations between variables and sought to identify significant predictors for the outcome variable –WPV. All tests were two-tailed, with the level of significance set as *p* < .05. Results were presented as odds ratios (ORs) and 95% CIs.

Depression, anxiety and stress scores were categorized into dichotomous responses (yes/no) before being submitted to univariate analysis. Participants with a cut-off score of ≥10 in depression, ≥8 in anxiety and ≥15 in stress dimension were considered as having these disorders as referenced by the DASS manual [30]. Statistical analysis was performed using SPSS Version 23.0 for the Windows platform (SPSS Inc.; Chicago, IL, USA).

## Results

A total of 850 nurses (female = 745) participated in the web-based survey, at a response rate of 5.3%.

### Socio-demographic characteristics of the participants

Most participants were female (87.6%), front line nurses (87.2%). The mean age of participants was between 34 and 44 years old (SD ± 2.79). Over half of the participants were married (55%) and almost all the remainder single (43%). Only a fraction were either divorced, separated or widowed (2%). Participants had an average of 10–20 years of clinical experience; 70% of them had obtained a Bachelor degree or above. Male nurses reported more WPV than their female counterparts.

Our gender ratio was 4: 1 (F:M) as against a wider gender ratio in the nursing population of Hong Kong was 7: 1 (F:M) [39]. A weighting was thus applied to readjust for gender before data were submitted to statistical analysis. The age, educational attainment and clinical experience of our respondents as a cohort were similar to those for non-respondents among the nursing population.

### Prevalence of physical and non-physical violence

A total of 44.6% (*n* = 379) of participants had encountered WPV in the preceding year. Verbal abuse/bullying (39.2%, *n* = 333) was the most common form of violence, followed by physical assault (22.7%, *n* = 193) and sexual harassment (1.1%, *n* = 9). Some participants had encountered more than one form of WPV. The most common perpetrators of WPV were patients (36.6%) and their relatives (17.5%), followed by colleagues (7.7%) and supervisors (6.3%). All participants were asked whether they thought the WPV they had suffered was preventable. Only 51.5% of participants so judged it.

Participants’ management of WPV included confiding in colleagues (31.2%), reporting incidents to supervisors/senior managers (29.6%), confronting the perpetrator(s) (13.4%) and ignoring incidents (9.2%). Only a small fraction of nurses requested a transfer (1.8%) or reported violence to their association (0.6%). 49.4% of the participants claimed incidents of WPV had a moderate impact on their mental health, while another 25.5% reported severe impacts.

### Binary logistic regression analyses

Table 1 reports the prevalence of WPV and its correlates. A total of 379 participants (44.6%) encountered WPV in the preceding year. Male nurses reported more WPV than their female counterparts (48.6% and 44%, respectively). Results showed a downward linear trend between age and WPV. As age increased, prevalence of WPV decreased. Younger nurses, in particular, those aged between 21 and 34, were at higher risk of experiencing WPV than older counterparts (cOR 3.04–3.26). Nurses with a bachelor degree or above were found to report more WPV than less-educated nurses. Mental health nurses were 0.6 times more likely than general nurses to experience WPV, and front line nurses 1.67 times to come across it than charge nurses. Nurses requiring shift work were 2.4 times more likely to report WPV than those on fixed day duty. Those dissatisfied with their jobs and those experiencing conflict with their colleagues were 1.88 times and 1.51 times more likely to experience WPV than those satisfied and not experiencing workplace disturbance. Nurses with a history of deliberate self-harm, depression, anxiety and stress symptoms (all *p*s < .05) were more likely than those without to report WPV (Table 1).

**Table 1:** Frequency distribution of respondents by workplace violence (WPV) and socio-demographic characteristics and other selected variables (*n* = 850)

Variables	WPV		*P*	cOR	95% CI	
	Yes (n)^c^	%			Lower Bound	Upper Bound
Sex						
Male^a^	51	48.6	-	-	-	-
Female	328	44.0	0.309	0.84	0.61	1.17
Age (Years)			**0.021**			
21–24	37	48.7	0.023	3.04	1.17	7.91
25–34	135	49.1	0.008	3.26	1.36	7.85
35–44	128	45.2	0.022	2.79	1.16	6.70
45–54^a^	71	38.2	0.120	2.04	0.83	4.99
Education level			**0.666**			
Bachelor degree or above^a^	271	45.5	-	-	-	-
Associate degree	47	43.5	0.575	0.89	0.59	1.35
Secondary school (Form 4–7)	61	41.5	0.430	0.86	0.60	1.25
Marital status			**0.309**			
Single, never married^a^	173	47.9	-	-	-	-
Married/cohabitant	196	41.9	0.130	0.81	0.61	1.07
Divorced/widowed/separated	11	50.0	0.986	0.99	0.41	2.39
Religion						
No	241	44.5	0.843	0.97	0.73	1.29
Yes^a^	137	44.6	-	-	-	-
Monthly household income (HKD)			**0.625**			
20,000–39,000	108	45.2	0.853	0.97	0.69	1.36
40,000–59,000	132	42.3	0.354	0.86	0.62	1.18
≥ 60,000^a^	139	46.6	-	-	-	-
Specialty						
General nursing	237	40.2	0.000	0.56	0.42	0.79
Mental health nursing^a^	142	54.6	-	-	-	-
Position						
Staff nurse	341	46.1	0.020	1.67	1.10	2.54
Charge nurse^a^	37	33.9	-	-	-	-
Years of employment						
< 10	189	47.0	0.183	1.20	0.92	1.58
≥ 11^a^	190	42.4	-	-	-	-
Shift work						
No^a^	73	29.6	-	-	-	-
Yes	306	50.7	0.000	2.44	1.78	3.35
Job satisfaction						
Dissatisfied	167	54.2	0.000	1.88	1.42	2.49
Satisfied^a^	212	39.0	-	-	-	-
Upset with colleagues						
No^a^	134	38.7	-	-	-	-
Yes	245	48.6	0.004	1.51	1.14	1.99
Chronic illness						
No^a^	279	42.3	-	-	-	-
Yes	100	52.6	0.012	1.51	1.09	2.09
Bereavement of first degree relatives in the past year						
No^a^	356	43.8	-	-	-	-
Yes	23	60.5	0.048	1.96	1.01	3.81
Bereavement of other relatives and friends in the past year						
No^a^	258	42.7	-	-	-	-
Yes	121	49.2	0.091	1.29	0.96	1.74
Exercise						
No^a^	334	45.4	0.214	1.29	0.86	1.93
Yes	45	39.5	-	-	-	-
Smoking status						
No smoking^a^	370	44.2	-	-	-	-
Smoking	9	75.0	0.057	3.38	0.97	11.85
Current drinker			0.487			
No^a^	282	43.5	-	-	-	-
Yes, 1–2 times/month	79	48.8	0.234	1.23	0.87	1.74
Yes, daily to few times/month	18	46.2	0.784	1.09	0.58	2.09
Entertainment						
No	246	46.9	0.088	1.28	0.97	1.69
Yes^a^	133	40.8	-	-	-	-
Maintain 7–8 h sleep 3–4 times/week						
No	258	47.2	0.037	1.35	1.02	1.78
Yes^a^	121	39.8	-	-	-	-
Current drinker			0.487			
No^a^	282	43.5	-	-	-	-
Yes, 1–2 times/month	79	48.8	0.234	1.23	0.87	1.74
Yes, daily to few times/month	18	46.2	0.784	1.09	0.58	2.09
Entertainment						
No	246	46.9	0.088	1.28	0.97	1.69
Yes^a^	133	40.8	-	-	-	-
Maintain 7–8 h sleep 3–4 times/week						
No	258	47.2	0.037	1.35	1.02	1.78
Yes^a^	121	39.8	-	-	-	-
Psychiatric disorder						
No^a^	372	44.2	-	-	-	-
Yes	7	77.8	0.064	4.94	0.91	26.76
Self-perceived physical health						
Poor	249	48.8	0.003	1.54	1.16	2.03
Good^a^	130	38.2	-	-	-	-
Self-perceived mental health						
Poor	191	51.8	0.000	1.67	1.27	2.20
Good^a^	188	39.1	-	-	-	-
Deliberate self-harm						
No^a^	334	43.3	-	-	-	-
Yes	45	57.0	0.019	1.75	1.10	2.75
Depressive symptoms						
No^a^	225	41.2	-	-	-	-
Yes^b^	154	50.5	0.009	1.46	1.10	1.94
Anxiety symptoms						
No^a^	216	40.4	-	-	-	-
Yes^c^	163	51.6	0.002	0.64	0.48	0.84
Stress symptoms						
No^a^	207	41.3	-	-	-	-
Yes^d^	172	49.3	0.023	0.73	0.55	0.96

*cOR* Crude odds ratio. ^a^Reference group. ^b^DASS Depression Scores ≥ 10 (mild, moderate, severe, extremely severe); ^c^DASS Anxiety Scores ≥ 8 (mild, moderate, severe, extremely severe); ^d^DASS Stress Scores ≥ 15 (mild, moderate, severe, extremely severe)

Significant data are highlighted in bold

### Multivariate logistic regression

In the final model, six variables – clinical position, shift work rotation, job satisfaction, conflict with colleagues, deliberate self-harm and symptoms of anxiety – emerged as significant correlates of WPV (Table 2). The strongest correlate was shift work rotation (aOR 2.67), followed by job satisfaction (aOR 1.72) and deliberate self-harm (aOR 1.66). Front line nurses were 0.34 times more likely than charge nurses to encounter WPV (95% CI 0.25–0.48). Nurses going through workplace issues were 1.4 times more likely than those not to report WPV (95% CI 1.03–1.95). Last, nurses with anxiety symptoms were 1.5 times more likely to report violence (95% CI 1.08–2.03) (Table **2**).

**Table 2:** Multivariate logistic regression model predicting workplace violence among Hong Kong nurses

Variable	Categories	B	S.E.	Wald	*p*-value	aOR	95% CI	
							Lower B	Upper B
WPV								
Constant		−0.795	0.181	19.365	0.000			
Position								
	Staff nurse	−1.065	0.172	38.23	0.000	0.345	0.25	0.48
	Charge nurse^a^	-	-	-	-	-	-	-
Shift duty	No^a^	-	-	-	-	-	-	-
	Yes	0.978	0.171	32.56	0.000	2.658	1.90	3.72
Job satisfaction	No	0.542	0.158	11.792	0.001	1.720	1.26	2.34
	Yes^a^	-	-	-	-	-	-	-
Upset with colleagues	No^a^	-	-	-	-	-	-	-
	Yes	0.346	0.163	4.495	0.034	1.414	1.03	1.95
Deliberate self-harm	No^a^	-	-	-	-	-	-	-
	Yes	0.507	0.256	3.920	0.048	1.661	1.01	2.74
Anxiety symptoms	No^a^	-	-	-	-	-	-	-
	Yes	0.391	0.160	5.949	0.015	1.479	1.08	2.03

*aOR* adjusted odds ratio. ^a^Reference group

## Discussion

Our results reveal WPV remains a significant concern in Hong Kong healthcare settings. Many of our respondents had suffered verbal abuse. The pattern of WPV as committed by representatives of different groups mostly matched that reported by other local [17, 18, 26] and international studies [8, 11, 20, 40–42]. Nearly 45% of nurses had encountered physical or psychological violence the preceding year, and 13.3% had sustained an injury in these incidents. Nonetheless, compared to Kwok et al.’s [18] findings, our results suggest WPV is less prevalent than previously estimated, though still at an alarming level.

For this study, WPV can come from either an external (i.e. from patients and/or their families) or internal source (i.e. from colleagues and supervisors). As Hong Kong’s population continues to rise, nurses are taking care of increasing numbers of patients in clinical settings. Nurses can be short-staffed and have to deal with fractious colleagues. As increasingly sophisticated forms of technology spread among a lay public, patients may have higher expectations of the quality of care provided by nurses. Nurses will always be busy and operate under severe time constraints. Staff shortages inevitably lead to longer waiting times for consultation and treatment. Nonetheless, patients and their families may perceive any delay as intolerable [43]. Patients may easily get frustrated when acutely sick or in pain. When patients’ immediate requests cannot be quickly met (whether they are actually being neglected or not), they may direct their anger and discontent at nurses [20, 43]. They may then become verbally abusive, bullying or even violent. Thus nurses’ job demands may significantly predict the incidence of external sources of WPV [44]. Additionally, mistrust of and miscommunication with healthcare workers may contribute to violence [45].

Past research has highlighted some risk factors for violence, for example, being a man [46], clinical inexperience, being younger, being less-educated, clinical position and type of hospital setting [11, 24, 25]. Some other researchers, though, have associated a higher level of education with WPV. Researchers would seem to disagree on the antecedents of WPV.

Our results show that male nurses reported more WPV than female nurses; this finding is similar to that in published works by Albashtawy et al. [24]; El-Gilany et al. [45]; Camerino et al. [25]; Pai & Lee [11] and Muzembo et al. [46]. Some of the cultural expectations attached to masculinity may explain the gender bias in reporting WPV. Male nurses are socialized to play a masculine role, suggesting they are less likely to bow to others’ unreasonable abuse and criticism of their work [24]. Another speculation is that male nurses may feel uncomfortable at some level with the feminized ‘caring’ roles they are expected to fulfil, with the result they are more likely to interpret criticism from patients as abuse. More importantly, in Albashtawy et al.’s study, violence against women in Jordan is culturally and religiously proscribed and may be thought of as less common.

A similar study [25] also found that male nurses disproportionately reported violence from patients and their relatives. Camerino’s findings support our speculation that male nurses may suffer from cultural assumptions about gender that may earn them less workplace respect and so alienate and frustrate them. These factors may aggravate WPV towards male nurses. Equally, men in Hong Kong, like in Jordan, may be disproportionately the objects of physical abuse and violent objections to their treatment from patients.

Front line nurses were more susceptible to WPV than charge nurses in being directly involved in patient care, rather than carrying out managerial or administrative tasks. Front line nurses also dealt immediately with enquiries and complaints. Ineffective communication between healthcare providers and care recipients may increase the risk of verbal abuse or bullying.

Participants educated to baccalaureate degree level report more WPV than those without degrees. In our sample, the 70% of participants with a baccalaureate degree or above may be less disposed to tolerate bullying and so less likely to suffer WPV in silence. Another theory would be that nurses with better academic training may have been less exposed to WPV in the clinical setting and less skilled in heading it off. Equally, this clinical inexperience may lead these better-educated nurses to report incidents to superiors in asking for advice. Our results were consistent with Jiao [20], though not in line with Albashtawy et al., [24], Camerino et al. [25] and Pai & Lee [11].

Interestingly, shift work also emerged as a significant correlate of WPV. Our study is limited by its cross-sectional data and so unable to investigate the causal relationship between shift work and WPV, nor single out any particular shift (morning, afternoon or night) during which nurses are more vulnerable to WPV. The Hospital Authority – arguably the largest healthcare organization in Hong Kong – categorizes shifts into morning, afternoon and night. In our sample, a significant majority of participants (70.9%) were on shift duty rotation. Those allocated a less desirable pattern (e.g., night duty) may be more likely to interpret this poor shift duty roster as a kind of bullying exercised by their supervisors [21]. In Hong Kong, morning shifts tend to be busier than afternoon and night shifts because consultant rounds (except urgent medical requests and referrals) take place in the mornings. WPV is more frequent when fewer nurses are working on the ward (e.g. during breakfast or meal breaks). Understaffing may be perceived as a lack of attention to patients’ immediate needs and may provoke abuse or hectoring from patients or relatives [44]. Recent research findings also find a positive relationship between morning shifts and bullying (β = .08, *p* < .001) [21]. Morning shift workers were more likely to experience bullying than other shift workers.

Our results also indicated that upset with colleagues was another significant correlate of WPV. Nursing demands that all qualified nurses can work independently and competently on their own and work as a team. Nonetheless, the health authority in Hong Kong has zero tolerance of any slips which endanger patient care. Any misconduct or negligence could also provoke a public outcry fanned by extensive media coverage. In this environment, nurses may be less eager to help colleagues, wanting rather to avoid blame and to protect their license to practise. Lack of team cohesion, low staff morale and conflicts of interest may stoke tension, lead to workplace conflict and lower the quality of patient care. In consequence, nurses are becoming even more liable to internal and external sources of workplace abuse.

Anxiety was also significantly correlated with WPV in nurses. Our findings were consistent with Jiao et al. [20, 47] in suggesting some personality traits among nurses may aggravate WPV. Specifically, negative affectivity (NA) may be considered a potential antecedent of bullying at work [40]. NA refers to an individual’s level of pervasive negative emotionality and self-concepts [48]. Individuals with NA may perceive others’ behaviours as more personal (or personally critical) than they actually are [49]. Individuals with NA will be disproportionately distressed by interpersonal conflict. They may understand conflict as destructively personal, experiencing an intensification of negative, stressful emotions. There may be a mechanism whereby people with anxious personalities may be more likely to get into interpersonal conflicts at work due to their acting in professionally incompetent ways. Patients often fear anxious nurses will make medical errors in their care – and abuse these carers accordingly. Some individual nurses may actually be anxious because they were previously victimized in the workplace and developed trauma. If these victims’ negative emotions are not addressed, their cumulative levels of anxiety may affect their job performance. Poor performers will clash with colleagues and attract further psychological WPV in turn.

WPV may have negative consequences for the victim’s health [50]. Nearly 75% of those affected in our study reported moderate to severe impact mental health impacts. WPV may also affect the victim’s job satisfaction and workplace confidence at work, accelerating emotional exhaustion and burnout [51, 52]. In extreme cases, sufferers may feel depressed, anxious or stressed at work [53]. In our sample, depression, anxiety and stress were significant correlates of WPV in the bivariate analysis; and anxiety emerged as a significant correlate in the multivariate analysis in the final model. Thus, the impact of WPV on nurses’ mental health cannot be under-estimated.

Horizontal violence happens when some nurses are submissive in their dealings with authority. This pattern of silence and submission may lead to feelings of fear, anger and low self-esteem, and the subsequent internalization on nurses’ part of aggressive behaviours and their redirection towards co-workers [54]. This study also found incidents of horizontal violence in the sense that the perpetrators of WPV were colleagues and supervisors within the healthcare setting. Recent research understands bullying not as a single, isolated event but repeated negative behaviour persistently manifested towards an employee [21]. More significantly, some nurses suffer WPV in silence because, it is strongly suggested, they do not think reporting WPV will change anything [55]. This attitude may result in under-reporting [56] or downplaying the seriousness of the negative impact of WPV on nurses’ mental health. Under-reporting indicates a lack of faith in the healthcare system responsible for preventing workplace violence.

Results also showed that deliberate self-harm is the third significant correlate of WPV in nurses. Past research has focused on the nature and perpetrators of WPV in healthcare settings without attending in comparable detail to nurses’ stress-coping mechanisms in processing WPV. Bivariate correlations showed that deliberate self-harm (DSH) was strongly associated with the DASS _stress subscore (*r* = .231, *p* < .001). It is plausible that some nurses have recourse to ineffective coping methods in reducing work-related stresses of WPV by self-harming, especially if they have not reported WPV to supervisors. Nonetheless, DSH also indicates impulsivity and aggression towards oneself. This finding is new in workplace violence research. Semi-structured qualitative interviews could be a feature of future studies examining causal links between DSH and WPV.

Past research has similarly taken little interest in the antecedents of WPV in healthcare settings. Studies consistently report nursing as one of the most vulnerable medical occupational groups to WPV, partly due to nurses’ direct contact with patients. Nurses have also been portrayed as exercising a limited scope of professional autonomy [54]. This low level of self-control and -responsibility may make nurses more susceptible to emotional abuse. Social support could, however, buffer the negative effects of WPV.

The Demands-Control-Support (DCS) model developed by Johnson & Hall [57] found that social support can diminish the negative psychological impact of high-strain jobs characterized by high demands and low levels of control. This DCS model is a widely used framework in explaining occupational stress (e.g., Baillien et al., [58]; Tuckey et al., [59]. Tuckey tested the DCS model on a sample of police officers, finding that increased job demands and a decreased level of control and available support resources were associated with more bullying at work. Nonetheless, there is a paucity of research using the DCS model to explain WPV in nursing, thus little is known whether it is generalizable to this context qua theoretical model. Faced with huge job demands and a narrow decision latitude, nurses may become socially isolated and behave like members of an oppressed group. Individuals without much support may be at higher risk of allowing internalized emotional abuse to fester.

In the local Hong Kong context, the Occupational Safety and Health Ordinance CAP 509 Section (6) requires that “every employer must, so far as reasonably practicable, ensure the safety and health at work of all the employer’s employees”. Depending on the gravity of the breach, failure to comply with this provision may result in financial penalties or imprisonment. Seven industries in Hong Kong have reported the greatest number of workplace violence incidents, of which medical and health-related services rank first. Under the ordinance, the Hospital Authority in Hong Kong could by bylaw prosecute perpetrators of WPV in court. Nonetheless, the bylaws are rarely enforced [17].

The International Labor Organization (ILO) and WHO have issued guidelines on the most effective ways to prevent WPV [6]. The European Union has also offered preventive advice on minimizing the threat posed by physical and psychological violence in the workplace [1]. England and Australia further adopt a zero tolerance approach to patients’ and visitors’ violence in hospital settings [2]. A survey commissioned by the Emergency Nurses Association (ENA) in 2006 (*n* = 1,000) found that the violence rates were significantly lower in hospitals (8%) with a zero-tolerance policy towards WPV than those facilities without (18%) [60]. Healthcare policy and legal enforcement thus have a significant deterrent effect on WPV.

The response rate of this study was relatively low (5.3%) compared with other similar local (7.7% and 25% respectively) [17, 18] and non-local studies (50% & 87%) [15, 16]. Voluntary questionnaires yield notoriously poor response rates. Nonetheless despite the low response rate, this study is exploratory in nature to estimate the weighted prevalence of WPV and its correlates towards nurses in Hong Kong. The authors assert that this is a reasonably good attempt to estimate the scale of the WPV problems before suggestion of effective WPV prevention strategies.

### Implications of this study

The first step in preventing WPV is encouraging a cognitive-behavioral change in nurses’ mindset, so that they no longer consider WPV a routine occupational hazard. Nurses should be proactive in reporting every single WPV incident to their supervisors/line managers to prevent similar incidents occurring. The health authority should also establish a highly transparent, user-friendly reporting system for front line nurses for WPV. Anti-violence protocols should be easily accessible to healthcare workers. Victims of WPV should be assured that incidents will be seriously and promptly dealt with by senior managerial staff. Stakeholders or healthcare providers should seriously consider adopting a zero-tolerance policy towards WPV towards healthcare workers or else risk the frustration, exhaustion, burnout, reduced job satisfaction, poor morale or, at worst, developing psychiatric morbidity of healthcare workers. Failing to act on WPV risks compromising the quality of patient care. The nursing curriculum of one local university now incorporates a module on the management of aggression. This programme, though, is only offered to students of mental health nursing. The training programme should be extended to all nursing courses, including the general and psychiatric stream and to other local universities.

In Hong Kong, where workplace violence is a common feature of psychiatric units, a working group was formed in a major local psychiatric hospital, leading to the development of an integral programme [26]. The primary goal was to prevent both patient and staff injury due to violence, to develop a harmonious clinical environment, and build up trust and respect between patient and staff. Newly recruited staff were required to attend the ‘management of violence’ workshops before working with psychiatric patients. Other regular staff had to attend the mandatory ‘psychiatric emergency drills’ to keep their knowledge up to date and become adept in using de-escalation skills. Most importantly, staff feel empowered and supported to respond appropriately to violent incidents in clinical settings.

## Conclusions

WPV seems to be a significant occupational hazard for the Hong Kong nursing profession. Healthcare providers and workers should jointly propose a cogent strategy of WPV prevention in healthcare settings. Nurses deserve a safe working environment free of physical, psychological or sexual abuse.
